# Postoperative multimodal pain management: a narrative review of current practices, clinical and educational gaps, and future directions

**DOI:** 10.3389/fanes.2025.1709252

**Published:** 2025-12-18

**Authors:** Braden M. Lopez, Brent M. Lee, Michael D. Miller, Mohab M. Ibrahim, Todd W. Vanderah, Arthur C. Riegel

**Affiliations:** 1 Creighton University School of Medicine, Phoenix, AZ, United States,; 2 Rocky Vista College of Osteopathic Medicine, Parker, CO, United States,; 3 Department of Orthopedic Surgery, College of Medicine, University of Arizona, Tucson, AZ, United States,; 4 Department of Anesthesiology, College of Medicine, University of Arizona, Tucson, AZ, United States,; 5 Department of Neurosurgery, College of Medicine, University of Arizona, Tucson, AZ, United States,; 6 Comprehensive Center for Pain and Addiction (CCPA), University of Arizona, Tucson, AZ, United States,; 7 Department of Pharmacology, College of Medicine, University of Arizona, Tucson, AZ, United States,; 8 Department of Neuroscience, College of Science, University of Arizona, Tucson, AZ, United States,; 9 James C. Wyant College of Optical Sciences, University of Arizona, Tucson, AZ, United States

**Keywords:** medical education, objective pain measurement, opioids, pain, postoperative pain management, surgical optimization

## Abstract

Pain is among the most commonly reported side effects following surgical interventions; however, its management remains a significant challenge due to its multifaceted nature, with studies indicating that up to 80% of surgical patients experience inadequate pain control. Although multimodal pain management (MMPM) is widely recommended as a tool to help mitigate the ongoing opioid epidemic, a universally standardized approach for pain management is lacking and highly dependent on individual clinician practices. Pain perception is inherently subjective, and while objective measurement tools are emerging, self-reported pain scales continue to dominate clinical practice. Differences in pain perception, further complicate efforts to standardize care, demonstrating the need for personalized approaches. Notably, there is a deficiency in surgical education regarding formalized training in postoperative pain management, which leaves medical students and residents without a concrete foundation in evidence-based pain management strategies. This narrative review explores the pathophysiology of pain, evaluates current recommendations in surgery, and emphasizes preoperative optimization. It also argues for, and underscores the necessity for, comprehensive and structured pain management education across all surgical specialties. Furthermore, the review identifies future directions, particularly in pain prediction and the development of surgical guidelines that can facilitate a comprehensive pain management framework while accommodating patient-specific modifications.

## 1 Background

### 1.1 The challenge of postoperative pain management

Effective management of postoperative pain is a clinical imperative, critical for enhancing recovery, reducing complications, and improving patient quality of life. While frameworks such as Enhanced Recovery After Surgery (ERAS) protocols have successfully reduced opioid consumption and standardized some aspects of perioperative care, the fundamental problem of pain persists (1). A startling proportion of patients, reportedly up to 80%, still experience inadequate pain relief following surgery (2). This failure in pain management is not a trivial matter; it is directly linked to significant adverse outcomes, including delayed ambulation, impaired wound healing, diminished functional recovery, and prolonged hospitalizations (3–7). The persistence of this problem suggests that simply having protocols is insufficient without addressing the complexities of their application.

### 1.2 Orthopedic surgery: a unique problem

The challenge of postoperative pain management is particularly important in orthopedic surgery. These procedures, designed to alleviate chronic pain and restore function, are themselves a source of significant acute postoperative pain. The cornerstone of recovery in orthopedics, early and progressive ambulation, is paradoxically limited by the very pain it aims to overcome (8–10). Furthermore, the pharmacologic agents used to control this pain can present a clinical dilemma. Two mainstays of pain management, opioids and non-steroidal anti-inflammatory drugs (NSAIDs), have been historically associated with impaired bone healing, a devastating complication in the orthopedic patient population (11, 12). However, recent meta-analyses indicate that this risk is largely dose-dependent and prolonged. Wheatley et al. demonstrated that short-course, low-dose NSAID use does not significantly increase non-union rates in adults, suggesting the therapeutic window for these agents is wider than traditionally taught. Not considering these points may culminate in a narrower therapeutic window, demanding a nuanced and evidence-based approach to balance pain control with optimal surgical outcomes.

### 1.3 The gap between principle and practice: a deficit in education

In response to these challenges, multimodal pain management (MMPM), the use of two or more analgesic agents or techniques targeting different pain pathways, has emerged as the standard of care (7). MMPM aims to provide superior pain relief while minimizing opioid-related adverse events, which occur in up to 11.5% of postoperative patients (13–15). The American Society of Anesthesiologists’ (ASA) 2021 consensus statement outlined seven principles that should guide acute perioperative pain management. Briefly, the seven principles are a thorough medical history taking, use of validated pain measurement tools, use of both pharmacological and non-pharmacological methods of analgesia, patient-centered education on pain management, educate patients on their specific treatment plan, adjust pain therapy as needed, and consult a pain specialist when symptoms are inadequately controlled (16). The overview is well-stated and establishes the foundational standard of care. However, while the ASA provides the framework, the granular application of these principles requires a constant re-evaluation of emerging pharmacologic data. This review aims to operationalize these consensus principles by synthesizing the most recent evidence on their practical implementation.

The principle of MMPM is far from its universal practice. A standardized, evidence-based approach is conspicuously lacking, leading to high variability in care that is dependent on individual clinician habits rather than established best practices. We argue that a primary driver of this gap is a systemic deficiency in formalized pain management education. This educational deficit represents a direct failure to meet the competency standards set by the ASA 2021 Guidelines (Principle 5), which explicitly mandate that clinicians provide comprehensive education to all patients regarding their pain treatment plan. A lack of structured training leaves clinicians without a foundational framework for implementing effective, personalized, and opioid-sparing pain management regimens.

### 1.4 Objectives of this narrative review

The discussions above highlight the complexity of postoperative pain control and a pressing need for protocol-driven frameworks that are both comprehensive and adaptable (17–19). Therefore, this narrative review seeks to address the educational and practical gaps in perioperative pain management. We will explore the underlying pathophysiology of pain, evaluate current pain management recommendations, and underscore the necessity for a structured educational curriculum. Furthermore, we will identify future directions in pain prediction and personalized medicine to propose a comprehensive framework that can guide clinicians in delivering optimal, evidence-based pain management.

## 2 Methods

### 2.1 Design and search strategy

This study was designed as a narrative review to identify foundational aspects of postoperative pain management, investigate emerging techniques, and identify current gaps in care. While a formal systematic protocol (e.g., PRISMA or MOOSE) was not applied, a structured literature search was utilized to ensure a comprehensive and objective overview.

Two authors (B.M.L. and A.C.R.) conducted the primary search using **PubMed** and **Google Scholar** between September 2024 and March 2025. The search strategy employed Boolean operators to capture concepts related to postoperative pain guidelines, multimodal analgesia, non-pharmacologic therapies, pain assessment, and educational gaps. Representative search phrases included: “*perioperative pain*” OR “*acute postoperative pain*” OR “*multimodal analgesia*” OR “*enhanced recovery*” OR “*opioid-sparing*” OR “*opioid-free anesthesia*.” Additional targeted searches were conducted to capture specific topics within orthopedic surgery, surgical education, and objective pain assessment technology.

### 2.2 Selection criteria

Inclusion criteria prioritized English-language publications from 2015 to 2025, ensuring the review reflected current practice. Earlier landmark studies were incorporated where necessary to provide historical or conceptual context. The review prioritized peer-reviewed clinical studies specifically randomized controlled trials, systematic reviews, meta-analyses, and scoping reviews. Clinical practice guidelines from major organizations, including the Centers for Disease Control and Prevention, American Society of Anesthesiologists, European Society of Anesthesiology and Intensive Care, and the American Medical Association, were also included.

Articles were excluded if they focused exclusively on chronic pain unrelated to surgery, pediatric populations (unless relevant to general principles), animal models, single-center case reports, or if full text was unavailable.

### 2.3 Article selection and synthesis

Initial broad database searches yielded a total of 86,189 records. To refine this pool to a manageable dataset relevant to adult perioperative care, automated filters were applied for publication date, language, and article type. Following these filters, approximately 11,389 records remained. Duplicate entries were removed.

Given the extensive volume of literature identified by broad search terms such as “pain” and “surgery”, the pool of 11,389 records included a significant number of studies unrelated to acute perioperative protocols (e.g., chronic pain management, non-surgical cohorts, and animal models). Therefore, a purposive selection strategy was employed to exclude these non-relevant themes and prioritize high-level evidence (Level I and II) specifically addressing: (1) pharmacological efficacy of multimodal pain management (MMPM) components; (2) gaps in surgical residency education regarding pain management; and (3) novel objective pain assessment technologies. Titles and abstracts were screened for relevance to these aims, acknowledging a degree of inherent subjectivity. Ultimately, 89 articles were selected for the final narrative synthesis to construct a representative overview of the current evidence-practice gap.

## 3 Core physiological and pharmacological basis of multimodal pain management

The pathophysiology of pain represents an intricate and multifactorial phenomenon encompassing several interrelated processes, which have been described in detail in several excellent resources (20–23). In brief, however, pain is at its core produced through a series of stages: transduction, transmission, modulation, and perception (24). Each of these stages involves a cascade of biochemical events, ranging from the initial activation of nociceptors to the final cortical processing of the pain experience (Table 1). Discussing the specific medications and techniques that target these distinct stages can provide a layered framework that helps illuminate why a single therapeutic intervention is often insufficient for effective pain control. In the context of transduction, noxious stimuli are first converted into electrical signals by specialized receptors located in peripheral tissues. The transmission phase of pain involves the propagation of electrical signals from peripheral nerves to the spinal cord and onward to the central nervous system. Modulation represents another critical stage in the pain pathway and largely involves complex neurochemical interactions within the central nervous system that adjust pain signaling. Finally, perception represents the subjective interpretation of pain and is influenced not only by sensory inputs but also by the patient’s emotional, cognitive, and psychological state. Table 1 details the specific pharmacological treatment that targets each stage of pain.

The interplay among the various stages of pain becomes particularly evident when considering the *limitations* of medications that target only one or two aspects of the pain cascade. For instance, while NSAIDs and acetaminophen are effective in reducing peripheral inflammation, they generally fail to modulate central pain processing adequately (23, 25–30). Similarly, opioids may broadly impact both the transmission and perception of pain (25, 28, 32, 33), yet excessive use can lead to long-term adverse outcomes such as dependency (14). Both tricyclic antidepressants (TCAs) and selective norepinephrine reuptake inhibitors (SNRIs) exhibit proven efficacy for chronic pain (34–37) yet require several weeks to reach therapeutic doses (38). These disparate factors highlight that any effective treatment of pain is likely best approached with an integrated treatment approach that combines multiple agents and techniques targeting several phases of the pain experience.

In summary, treatment of the different pathophysiological phases of pain, from transduction to perception, requires distinct yet complementary interventions. Failure to address any of these stages in a balanced manner can lead to suboptimal outcomes and may predispose patients to chronic pain conditions. Accordingly, the basic science behind pain transmission is not only relevant from a pharmacologic perspective but also holds critical implications for clinical practice. By fully appreciating the complex, multifactorial nature of pain, clinicians are better positioned to design effective multimodal regimens that are more responsive to both the sensory and emotional dimensions of sustained postoperative pain.

## 4 Multimodal pain management (MMPM): current practices

The primary aim of MMPM is to effectively manage and reduce pain by combining different types of medications and techniques, ultimately aiming to improve patient care, recovery time, and minimize opioid use. This approach seeks to achieve superior pain control with fewer side effects and a reduced risk of opioid dependence (17–19).

Within the multimodal paradigm, a variety of analgesic agents and techniques are employed simultaneously to target different stages of the pain cascade (Table 1). Common postoperative regimens in MMPM often include a combination of NSAIDs, acetaminophen, local wound anesthetics, anticonvulsants, systemic corticosteroids, muscle relaxers, and, when necessary, agents such as ketamine (39, 40). Ketamine is distinct and typically reserved for special cases, where it can be especially beneficial in opioid-tolerant patients or those with chronic pain conditions. However, its administration requires careful consideration due to potential side effects and contraindications (41). Importantly, the selection and dosing of these agents are tailored to both the type of surgery performed and the individual patient’s risk factors, including their history of previous pain experiences and their potential for adverse drug reactions (40). Notably, evidence suggests that while opioids are often indispensable for managing moderate to severe pain, other analgesics can be equally effective in the management of acute pain episodes (42, 43), potentially reducing reliance on opioids. This integrated approach underlines the clinical benefits of employing two or more agents with differing mechanisms of action, which is the cornerstone of modern multimodal pain management strategies.

### 4.1 Opioid-sparing analgesia vs. opioid-free analgesia

Within multimodal pain management, there are two distinct subsets, opioid-sparing analgesia (OSA) and opioid-free analgesia (OFA). There is mixed evidence that OFA is truly a feasible option for patients perioperatively and is highly dependent on specific surgeries. Intraoperatively, there is currently no strong evidence that OFA is superior to OSA in reducing hospital stay or postoperative opioid use (44, 45). Two studies found that OFA at discharge, for minor or moderative surgeries (as defined by the Operative Severity Score for the Enumeration of Mortality and Morbidity), did not lead to changes in patient reported satisfaction scores, but did decrease postoperative nausea and vomiting (PONV), constipation, and dizziness (46, 47). In orthopedic surgery specifically, there have been mixed findings, as some studies reported success with OFA while another reported that only six out of sixteen total joint replacement surgeries were able to maintain OFA without using opioids as a rescue pain medication (48, 49). In general, there has been significant heterogeneity found amongst OFA methods, both in our own observations and in the literature (50).

In the last 5 years, there have been multiple studies published that compare OSA to OFA all with differing results. What is leading to this discrepancy? Is it lack of education or perhaps the comfortability clinicians have of their current regimen? We believe that both of those contribute to the problem, but the key issue seems to be non-standardized plans for postoperative pain management across OFA methodologies. To our knowledge, there is only one 2025 study that compares OSA to OFA for different surgeries and include specific regimens (with drugs, dosing, and scheduling) across five common orthopedic surgeries (51). We conclude that a divergence exists between the physiological benefits of OFA and its clinical necessity. While studies like Pershad et al. demonstrate clear advantages in reducing PONV, the non-inferiority findings of Turk et al. regarding pain scores suggest that complete opioid elimination offers diminishing returns when compared to a rigorously optimized opioid-sparing protocol. This likely reflects a ‘ceiling effect’ where multimodal OSA is already highly effective. Consequently, OFA should perhaps be stratified for high-risk phenotypes (e.g., history of severe PONV or sleep apnea) rather than applied as a blanket mandate. The protocol in Turk, et al. is an excellent starting point for OFA and its findings must be expanded to address other surgeries both in and outside of orthopedic surgery, in order to establish detailed MMPM guidelines. These findings should continue to be tested in large, multicenter RCTs to further strengthen the rigor of standardized regimens. After the standardization of opioid-free analgesia regimens, then further steps must be taken to ensure adequate physician education and implementation occurs to reduce the use of opioids in the perioperative setting.

### 4.2 Critical re-evaluation of multimodal components

Recent high-impact studies reinforce the effectiveness of MMPM while challenging the necessity of its traditional components, underscoring how nuanced pain management regimens must be. In a recent analysis by Graham, et al., MMPM following non-cardiac surgery resulted in significantly reduced opioid use, highlighting the overall effectiveness of MMPM (52). This study found that the combination of NSAIDs with dexamethasone resulted in the greatest reduction in patient-reported pain and opioid use. While acetaminophen, a near universal cornerstone of MMPM, was shown to reduce postoperative pain in certain types of surgical procedures (53, 54) and even reduce hospital stay, it was not as efficacious as the combination of NSAIDs and dexamethasone in decreasing postoperative pain. This challenges the habitual inclusion of intravenous acetaminophen, especially considering its cost, when potent anti-inflammatory agents are utilized. Furthermore, because acetaminophen acts independently of inflammatory pathways, this finding highlights the paramount role of inflammation in driving postoperative pain, as both NSAIDs and corticosteroids directly target these pathways. The study’s limitations include its retrospective analysis of Veteran Hospitals exclusively, which skews the patient population toward older men, and its focus on inpatient management, where clinicians can adjust MMPM regimens with greater ease and precision compared to outpatient settings.

Beyond comparing the efficacy of individual components, the dosing and length of prescription for each drug demand critical consideration. A systematic review and meta-analysis by Laconi, et al. investigated the effects of high-dose dexamethasone (0.2 mg/Kg or ≥15 mg) at reducing postoperative pain (55). Beyond managing pain and inflammation, dexamethasone is highly effective at reducing PONV. The study found that high-dose dexamethasone administration reduced oral morphine equivalents by 10 mg and significantly reduced the odds of PONV (odds ratio 0.29). Interestingly, the study failed to identify a significant difference in patient-reported pain scores when using high-dose dexamethasone. The major limitation in applying these results clinically is the disparity between objective and subjective measures: while a reduction in opioid consumption is inherently beneficial for patient safety, the failure to reduce the patient’s perceived pain underscores the complexity of using opioid-sparing metrics over subjective comfort measures.

These studies emphasize the nuanced nature of effective MMPM. The selection of agents must balance analgesic efficacy against patient-specific risks. For instance, while NSAIDs and corticosteroids are highly effective, they can impair bony healing or neutrophilic response, which is a known contributor to chronic pain (56). In patients with certain renal diseases, NSAIDs may be unadvisable, forcing clinicians to lean more heavily on acetaminophen or opioids. Similarly, reducing postoperative pain with dexamethasone may require an increased dose, but this can significantly elevate blood sugar levels, presenting a possible unpredictable hyperglycemia in diabetic patients with the possibility of increased risk for operative site infection and delayed incision healing. Balancing standardized protocols with patient-specific individualization is difficult, yet spending additional time preoperatively to discuss confounding variables and properly plan an appropriate, tailored MMPM regimen is paramount.

### 4.3 Non-pharmacological options for MMPM

Adjunctive measures serve as an integral supplement to pharmacologic interventions under multimodal pain management. Beyond medications, techniques such as Transcutaneous Electrical Nerve Stimulation (57), Continuous Passive Motion (58), acupuncture (59, 60) psychological therapies (61, 62), and even emerging modalities such as cryoneurolysis (63) are being increasingly used to address pain (Table 2). Although these adjunctive strategies are not uniformly adopted across all clinical settings, their potential benefits in reducing reliance on opioids and improving patient outcomes have been thoroughly documented (64, 65) For instance, the employment of icing protocols, rest strategies, and physical therapy regimens has been shown to be instrumental in strengthening the musculoskeletal system and facilitating recovery (66). Additionally, it has been demonstrated that engaging in light, music, or olfactory therapy can reduce reported pain scores in chronic pain patients (67–72). Although these studies did not directly assess the efficacy of these techniques for postoperative acute pain, such approaches represent a potential tool for postoperative pain refractory to common pain management techniques. The inclusion of such techniques can help ensure that pain management is approached from a truly holistic perspective, addressing both pharmacologic and non-pharmacologic dimensions of postoperative management.

It should be emphasized that despite the growing consensus on the benefits of MMPM, there still remain considerable variations in the actual protocols employed by different surgeons and institutions. Such variations may be attributable to individual clinician training, differences in patient demographics, and institutional guidelines (73, 74). The guiding principle that opioids should be the last medication added and the first removed, as supported by clinical guidelines (75, 76), reinforces the need for a multimodal regimen that prioritizes functional outcomes over mere numerical pain scores. Likewise, immediate-release opioids are preferred over extended-release formulations, and opioid requirements should decrease as the patient recovers (77–80). Hence, it is incumbent upon clinical teams to continually update their practices in light of emerging research and guidelines.

In practice, the successful implementation of multimodal pain management requires that clinicians remain flexible and responsive to individual patient needs. Even when standardized regimens are in place, clinicians frequently need to adjust the specific components of the pain management plan based on factors such as patient comorbidities, intraoperative findings, and postoperative recovery profiles (76). Such adjustments may include the careful titration of non-opioid analgesics and the timely removal of opioids as patients progress along the postoperative timeline (76). By adopting such a responsive approach, clinicians can better ensure that changes in pain intensity and quality are swiftly addressed. As such, multimodal pain management is not static but rather represents a dynamic process that must adapt to the evolving clinical context.

**FIGURE 1:**
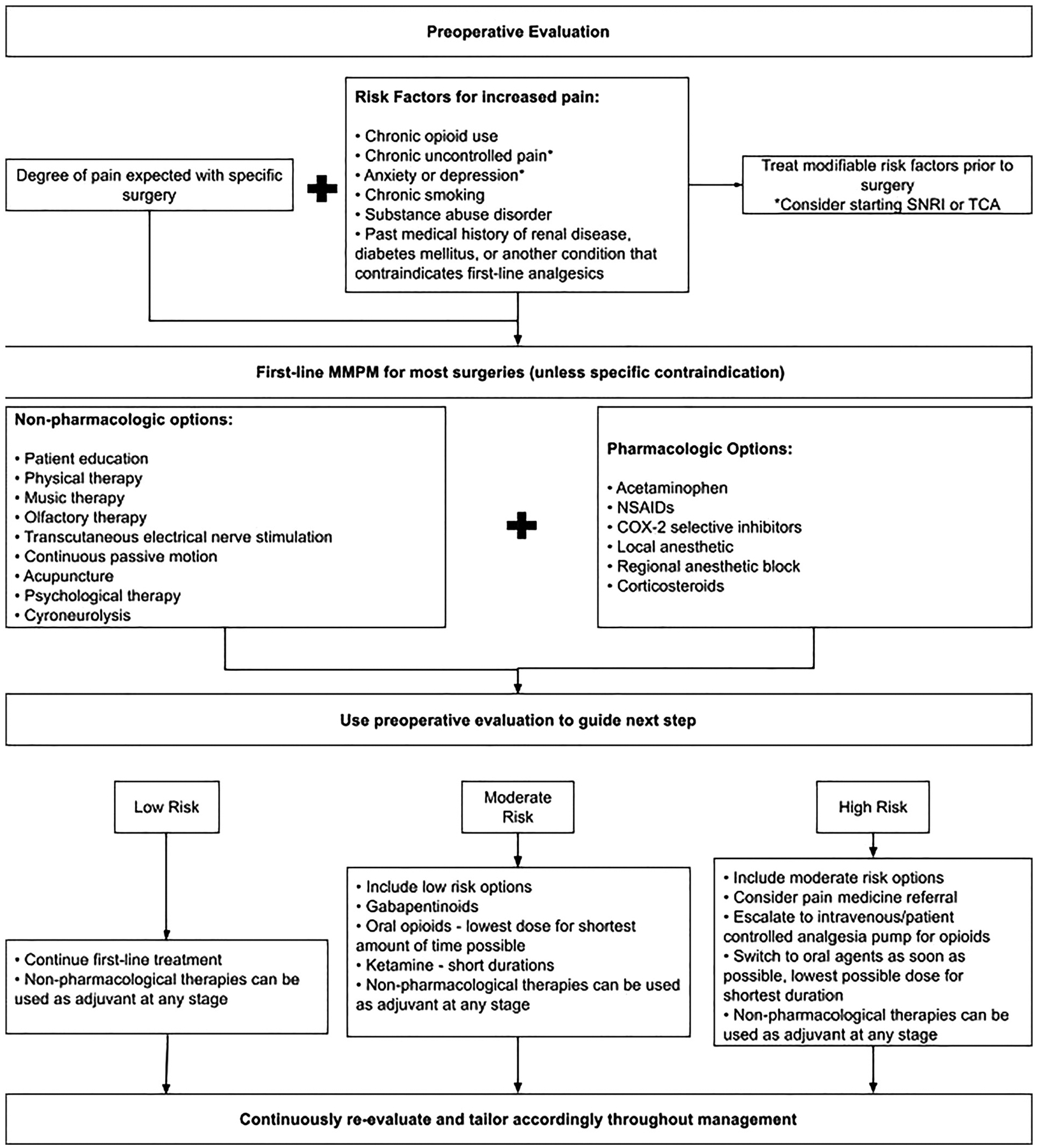
Schematic representation of the multimodal pain management (MMPM) strategy. This framework illustrates the synergistic combination of pharmacological interventions—including systemic agents (e.g., NSAIDs, Acetaminophen) and regional techniques (e.g., nerve blocks, local infiltration)—with non-pharmacological methods to target multiple facets of the neurochemical pain pathway. This integrated approach is designed to maximize analgesia and accelerate functional recovery while minimizing reliance on opioid medications.

Ultimately, the critical goal of multimodal pain management is to optimize the patient’s functional recovery (81) while minimizing the risks associated with analgesic overdose or dependence. By integrating multiple analgesic techniques, clinicians are better equipped to help reduce the overall drug burden and mitigate the adverse events that often accompany traditional opioid-centric approaches. Extensive data support the notion that multimodal regimens not only improve pain outcomes but also facilitate earlier ambulation, more rapid healing, and overall improved postoperative satisfaction (82). Figure 1 illustrates a stepwise approach to MMPM, where clinicians start with preoperative assessment, stabilize preoperative risk factors, start with the foundations of MMPM, then can modify care based on the risk calculation of the patient and specific operation (Figure 1). Given the considerable advantages of these strategies, it becomes imperative that clinical education and practice continue to emphasize the benefits of a multimodal approach. Thus, there remains a genuine need for patient-tailored protocols that incorporate both pharmacologic and nonpharmacologic techniques in pain management.

## 5 Preoperative optimization and risk stratification

### 5.1 Risk factors for increased postoperative pain

Postoperative pain outcomes are significantly influenced by preoperative risk factors that can be categorized as modifiable and non-modifiable (83). Non-modifiable risk factors such as age, sex, and inherent medical history are consistently shown to influence pain outcomes, and although these characteristics cannot be altered, they provide valuable context for anticipating pain severity (84, 85). Conversely, modifiable factors, including diabetes, chronic opioid use, and tobacco consumption (86), present opportunities to intervene prior to surgery, thereby reducing the potential for adverse pain-related outcomes. A careful and systematic preoperative assessment that encompasses these risk factors is critical for personalizing postoperative pain management plans.

The Centers for Disease Control and Prevention has long advocated for the careful evaluation of risks and benefits in pain treatment protocols, thereby encouraging a comprehensive preoperative optimization strategy (87). Initiating effective postoperative pain management at the time of the initial consultation is essential, as this allows for the identification of risk factors that may complicate recovery. The establishment of guidelines that prioritize preoperative risk stratification is paramount in minimizing the incidence of uncontrolled postoperative pain (88). These risk-based approaches also help to inform surgeons’ decisions regarding the appropriate pain management regimen and potential modifications to expected recovery timelines.

In clinical practice, the adjustment of modifiable risk factors prior to surgery can translate into measurable improvements in postoperative recovery (89). For instance, maintaining hemoglobin A1c levels below a critical threshold in diabetic patients has been shown to improve surgical outcomes and reduce postoperative pain intensity (90). Similarly, the practice of encouraging patients to cease tobacco use for at least four weeks prior to surgery is supported by studies demonstrating reduced postoperative complications and a more rapid recovery trajectory (91, 92). The preoperative management of medications, including the establishment of clear guidelines for opioid weaning, further contributes to improved pain outcomes (93–95). Consequently, a comprehensive evaluation that incorporates both modifiable and non-modifiable risk factors forms the cornerstone of successful postoperative pain management strategies. It is important to note that preoperative optimization is not completely possible in emergent surgeries or some cases. Many patients do adhere to their medical management yet may not reach complete optimization prior to needing surgical intervention. These are guidelines, and clinicians and surgeons should use preoperative optimization as much as possible, while also relying on clinical experience to inform decisions.

When contemplating preoperative risk factors, it is essential to recognize that the existence of chronic pain conditions significantly predisposes patients to more severe and persistent postoperative discomfort. Chronic back pain, for example, is a prevalent condition that can complicate postoperative pain management, particularly in orthopedic procedures (96). The presence of such conditions necessitates a thorough review of prior pain management regimens and, in many cases, the reevaluation of pharmacologic strategies prior to surgery.

Mental health plays a significant role in pain perception, as patients with anxiety and depression consistently report higher levels of postoperative pain. Numerous studies have documented that individuals with pre-existing psychiatric conditions, particularly those suffering from anxiety and depression, tend to report higher levels of postoperative pain compared to patients without such conditions (97). This stark relationship underscores the necessity for preoperative psychiatric screening as an integral element of the overall pain management strategy. The literature has documented that even brief preoperative educational interventions that address psychiatric concerns can have a measurable impact on postoperative pain outcomes (98, 99). It has been demonstrated that perioperative psychological interventions targeting anxiety can significantly reduce postoperative pain in elective abdominal surgery, further reinforcing the link between preoperative anxiety and poorer perioperative outcomes (100). This discrepancy calls for concerted efforts to better integrate psychiatric evaluations and educational interventions into the standard preoperative workflow. We recommend referral to a primary care or psychological clinician in order to achieve appropriate psychiatric stabilization, however, we also challenge surgeons to be comfortable prescribing the proper pharmacological intervention for their patients, as this preserves continuity of care.

In the context of preoperative risk assessment, the interplay between biological, behavioral, and psychiatric factors demands that clinicians adopt a multidimensional evaluation strategy. Although non-modifiable factors such as age and sex provide a baseline understanding of patient risk, modifiable factors such as diabetes, opioid use, and tobacco use offer actionable targets for intervention (86, 87). Lastly, it has been well documented in recent studies that proper preoperative patient education on the realistic expectations of postoperative pain improves patient satisfaction, especially in the first week postoperatively (64, 101). The refinement of these assessments through validated screening tools and evidence-based protocols coupled with proper patient education holds the potential to substantially mitigate postoperative pain and its associated complications. By integrating these diverse risk factors into a single, coherent preoperative evaluation system, clinicians can enhance both real-time decision-making and long-term outcomes. This holistic approach underscores the importance of a preoperative strategy that is both systematic and individualized.

### 5.2 Advances in pain assessment

In addition to screening for psychiatric risk factors, advanced pain assessment strategies are increasingly being explored as a means of more objectively quantifying the complex pain experience. Recent advancements in pain measurement offer the opportunity to transcend the limits of traditional subjective scales. Although conventional tools like the Numerical Pain Rating Scale (NPRS) and Visual Analog Scale (VAS) remain in common use (102, 103), their reliance on self-report leaves room for ambiguity (104). Alternative techniques such as pupillometry, functional near-infrared spectroscopy (fNIRS), and wearable forehead sensors have emerged as potential adjuncts that offer more objective correlates of the pain experience (105–107). These novel modalities are gradually being integrated into clinical research, promising to enhance our overall understanding of pain physiology and providing a more nuanced approach to postoperative pain management.

Advanced multimodal approaches in pain assessment further include neurophysiological tools like electroencephalogram (EEG) and functional magnetic resonance imaging (fMRI), both of which allow for direct visualization of brain activity associated with pain processing. Although the application of these sophisticated techniques in routine clinical practice is currently limited by factors such as cost, training requirements, and time constraints, they nonetheless represent promising avenues for future research (108). When used in conjunction, these tools may eventually facilitate the development of reliable “postoperative pain predictor” models that can be applied on an individual basis. These developments underscore a pivotal shift towards integrating objective pain measurements into the broader framework of pain management. These new objective techniques for measuring pain are summarized in Table 3.

Addressing limitations inherent to traditional pain scales, such as the NPRS and VAS, is critical for the development of more precise postoperative risk calculators (109). Although these scales remain valuable for their rapid and cost-effective nature, baseline measurements of patient pain tolerance are seldom obtained, thus potentially obscuring their reliability. The integration of alternative validated tools, including the Functional Pain Scale (110) and the Brief Pain Inventory (111), offers additional layers of context by assessing the impact of pain on patient function. Nevertheless, these instruments are also fundamentally subjective and must be interpreted within the broader clinical picture. There is a strong argument that pain measurements *should* primarily be subjective, given that pain is inherently subjective in its experience. Objective measures may be difficult to accurately apply across diverse populations because pain is often more than a purely biological phenomenon. As objective pain measurement technologies advance, it is essential to maintain cultural humility and respect for diversity, especially when applying these tools to groups that have historically faced health inequalities. With continued research, refinements to these assessment modalities may ultimately lead to more robust, individualized risk stratification systems in the perioperative setting.

### 5.3 Preoperative risk calculation

The evolution of pain assessment technologies also highlights the importance of ongoing research and validation in the realm of clinical pain measurement. As new studies emerge, a critical goal lies in refining these techniques so that they can be seamlessly integrated into existing clinical work1ows without imposing excessive burdens on healthcare resources. In doing so, new researchers aim to strike an optimal balance between technological sophistication and routine applicability, ultimately establishing a new standard for pain measurement. The development of operation-specific “postoperative pain predictor” tools could provide surgeons with invaluable data that correlates specific surgical interventions with quantifiable pain measures. Such a tool would potentially allow for the individualized risk stratification of patients, thereby facilitating tailored analgesic regimens that preemptively address potential pain complications. The eventual success of these endeavors will depend on rigorous clinical evaluations that substantiate the reliability and reproducibility of these novel approaches.

## 6 A greater role for pain education

The educational component of pain management further encompasses the need to train all surgical specialties on advanced strategies for postoperative pain management, including when to incorporate multimodal techniques and how to tailor these regimens to individual patient profiles. Studies have indicated that the majority of information regarding medication prescribing is obtained from attending surgeons (112), resulting in considerable variability among instructors and, subsequently, among new clinicians. For new residents and interns, a foundation in pain management is critical, as insufficient training in this area has been linked to suboptimal prescribing practices and increased discomfort for patients (113). To address these gaps, medical schools and residency programs should incorporate comprehensive training modules that emphasize both theoretical knowledge and practical, case-based learning in pain management, as was recently argued in an American Medical Association Journal of Ethics opinion (114). Such initiatives may include simulation-based modules, extended rotations in surgical palliative care, and mentorship programs that foster best practices. Furthermore, one study found that adherence to standardized order sets and focusing on ERAS principles in surgical residents led to shortened hospital stays and favorable patient outcomes after surgery (115).

This urgency is reinforced by the 2023 Pain and Opioids after Surgery consensus statement from the European Society of Anaesthesiology and Intensive Care, which used a modified Delphi survey to evaluate barriers to opioid-sparing MMPM (116). While panelists strongly supported the efficacy of MMPM, they identified the lack of training and education and the reluctance to change existing practices as the most critical barriers to widespread adoption. These findings refocus the necessary effort from overcoming logistical hurdles, such as cost and workload, toward systemic educational reform.

Despite recent efforts to integrate comprehensive pain management curricula into medical education, concerns persist regarding the adequacy of current training protocols, particularly in the domain of opioid prescribing. Studies have indicated that a significant proportion of surgical residents feel inadequately prepared to manage postoperative pain (117), a finding that underscores the need for ongoing curricular refinements. The Association of American Medical Colleges has argued for more foundational training in opioid and addiction in medical school training (118). Boot camp–style courses in pain management have been recommended as a means of bolstering confidence and competence among newly graduated physicians. Such educational innovations not only improve resident comfort levels but may also translate into enhanced patient outcomes through more judicious and effective use of analgesics (119). While broader curricular reforms, such as condensed medical education timelines, are gaining attention, we argue that focused, specialty-specific training in multimodal perioperative pain management presents a more immediate and impactful target for educational innovation. This evidence-based push for enhanced pain education reinforces the importance of a well-rounded clinical training program that addresses both pharmacologic and non-pharmacologic modalities. We have previously argued that education is a major factor preventing the complete implementation of opioid-free analgesia (OFA). Changing established clinical guidelines requires addressing preconceived ideas by starting at the foundation. While OFA is unlikely to be a heavily tested concept on licensing examinations, the topic is essential for real clinical practice. As medicine continues to evolve and testing material becomes more comprehensive and difficult, medical schools and residency programs must find the ideal balance between test preparation and clinical preparation, ensuring learners acquire the newest information necessary for the best patient care.

In addition to resident training, it is also incumbent upon practicing surgeons to engage in continuous professional development with regard to the latest advances in pain management. The field is rapidly evolving, with emerging therapies and objective measurement tools reshaping the clinical landscape. Attending surgeons, whose practices often set the standard for institutional protocols (113), must therefore remain committed to ongoing education and innovation. This commitment to lifelong learning will allow for the timely incorporation of new evidence into clinical practice, ultimately benefiting both patients and the broader healthcare system. Ensuring that all clinicians, regardless of their career stage, receive ongoing training in pain management is essential for the sustained success of multimodal pain management strategies.

## 7 Conclusion

The importance of tailoring pain management strategies is further highlighted by data indicating that individualized approaches yield superior outcomes compared with one-size-fits-all protocols (120–122). Each patient’s unique background, including physiological, psychological, and social histories, influences their response to both surgical stress and analgesic interventions. As such, the development of individualized pain assessment tools, potentially aided by objective measurement techniques, could prove invaluable in constructing a truly personalized pain management plan. By leveraging detailed preoperative evaluations, including both quantitative and qualitative assessments of pain, surgeons can more accurately predict the risk of postoperative complications and adjust treatment strategies accordingly. Such precision in pain management may ultimately pave the way for the next generation of tailored surgical care.

An effective pain management strategy must extend beyond pharmacology. While robust multimodal regimens remain foundational, future research should prioritize the integration of innovative objective measures of pain into comprehensive risk assessment tools. These advancements may include wearable sensor technologies, advanced neuroimaging modalities, and other novel approaches that provide real-time feedback on a patient’s pain status. The development of a postoperative pain predictor tool stands as a promising prospect, one that may eventually correlate specific surgical interventions with objective pain indices. Such innovation is critical not only for advancing clinical research but also for improving real-world outcomes and optimizing care.

It is important to recognize that postoperative pain management is nuanced. Coordination among multiple specialties and the influence of insurance reimbursement structures frequently complicate treatment approaches (123). Ideally, all clinicians would personalize analgesic regimens using a patient-centered framework. However, in practice, personalization is often limited by factors such as time constraints, technological availability, and institutional resources. Consequently, true precision in pain management is frequently underutilized.

Recommendations for future clinical practice include the development of evidence-based guidelines that integrate both subjective pain reports and objective measures into a cohesive postoperative pain assessment framework. The ultimate goal of such guidelines would be to provide surgeons with a robust, patient-specific risk calculator that encompasses surgical factors, medical history, and objective pain metrics. With such tools in place, clinicians would be better equipped to tailor multimodal analgesic regimens proactively and responsively. An individualized postoperative “pain-risk calculator”, grounded in comprehensive clinical data, has the potential to redefine perioperative pain management and serve as a cornerstone of optimized care. Additionally, psychological assessment and perioperative mental health interventions address a key gap: managing anxiety not only improves pain outcomes but also aligns with enhanced recovery protocols that emphasize holistic optimization (124–126).

In clinical practice there are several main takeaways from this narrative review. Where possible, surgeons are encouraged to conduct thorough preoperative screening for chronic conditions, substance use, and psychiatric history, all of which are critical for developing an individualized pain management plan. There is ample evidence that preoperative optimization leads to significant decreases in postoperative pain, thus decreasing patient discomfort, medical expenses, and clinical burden. Lastly, as clinicians adopt routine evidence-based multimodal protocols, the integration of patient education and risk-based adjustments will become indispensable. Such a comprehensive framework underscores the ongoing need for collaboration among all members of the surgical care team.

## 8 Future directions and research gaps

To address the current research gaps, future studies must prioritize innovation that enables personalized medicine. Specifically, research should focus on the utility of Artificial Intelligence and machine learning to analyze preoperative patient data (comorbidities, psychiatric history, opioid use) alongside intraoperative variables (procedure type, anesthetic technique, inflammatory markers) to generate a personalized perioperative risk prediction score. Such an artificial intelligence-driven approach would facilitate the creation of truly tailored anesthesia protocols, moving beyond broad guidelines to recommend procedure- and patient-specific dosing and medication combinations in a manner that maximizes opioid-sparing efficacy and functional recovery. Closing the gap between evidence and practice also requires large-scale, international implementation studies that specifically test novel educational interventions designed to overcome clinician inertia and institutional barriers.

## 9 Limitations

This narrative review is subject to several limitations. As it is not a systematic review, there was no pre-registered search strategy or formal methodology, which may introduce selection bias and limit reproducibility. The broad scope of the topic, encompassing pharmacologic, non-pharmacologic, educational, and technological domains, inherently introduces heterogeneity, making it difficult to draw uniform conclusions or propose standardized protocols. Lastly, we focused on the training in residency and medical school, specifically focused on postoperative pain management, which inadvertently steered our attention toward the initial training received, rather than a more comprehensive exposure necessary for diverse clinical experiences.

## Figures and Tables

**TABLE 1:** Analgesic mechanisms of action. This table details the primary physiological pathways (e.g., cyclooxygenase-inhibition, N-methyl-D-aspartate-receptor antagonism, sodium channel blockade) for key pharmacological agents used in a multimodal pain management regimen.

Pain sensation modality	Effective pharmacologic therapies
Transduction (23–30)	NSAIDs, acetaminophen, local anesthetics, Cyclooxygenase-2 selective inhibitors, and corticosteroids
Transmission (25, 30, 31)	Opioids, local anesthetics, anticonvulsants, and antispasmodics
Modulation (25, 28, 32, 33)	Opioids, TCAs, SNRIs, ketamine, and anticonvulsants
Perception (25, 28, 33, 34)	Opioids, general anesthetics, TCAs, SNRIs, and benzodiazepines

**TABLE 2:** Comparative table of Key pharmacological and Non-pharmacological interventions in MMPM. This table summarizes and compares evidence-based pharmacological and non-pharmacological interventions, their proposed mechanisms, and typical applications in the perioperative setting. This list is not exhaustive, but covers the most common options available to clinicians.

Intervention	Indications/clinical role	Reported outcomes	Limitations/key considerations
NSAIDs (7, 11, 23, 28, 42, 43, 45, 48)	Foundational MMPM component. Targets inflammation, a primary driver of postoperative pain.	High efficacy. In combination with dexamethasone, showed the “greatest reduction in patient-reported pain and opioid use”.	Contraindicated in renal disease. Can impair bony healing, a significant concern in orthopedic surgery.
Acetaminophen (7, 23, 26, 42, 45–48)	A “near-universal cornerstone of MMPM”.	In one major study, “did not reduce postoperative pain as significantly” as the NSAID + dexamethasone combination.	Does not target inflammatory pathways. Its habitual inclusion is being challenged, especially considering the high cost of IV formulations.
Corticosteroids (e.g., Dexamethasone) (7, 19, 30, 45, 48)	Manages postoperative pain and inflammation. “highly effective at reducing postoperative nausea and vomiting (PONV)”.	High doses (≥15 mg) “reduced oral morphine equivalents by 10 mg” and “significantly reduced the odds of PONV”.	May not impact *patient-reported pain scores* despite reducing opioid use. Can “significantly elevate blood sugar levels,” (a danger for diabetics) and potentially impair bony healing.
Opioids (12, 18, 25, 32, 42, 43)	“Indispensable for managing moderate to severe pain”. Guiding principle: “last medication added and the first removed”.	Effective for acute pain, but other analgesics can be “equally effective” in some episodes.	High risk of adverse events (e.g., PONV), dependency, and complications. Can potentially impair bone healing.
Transcutaneous Electrical Nerve Stimulation (TENS) (50)	Used to address postoperative pain, neuropathic pain, and chronic conditions.	“potential benefits in reducing reliance on opioids” when compared to placebo	“Not recommended for continuous use”. Can lead to tolerance and reduced movement.
Continuous Passive Motion (CPM) (51)	Utilized to “increase joint mobility and prevent stiffness” postoperatively.	A recent study found CPM rehabilitation was associated with increased ROM, pain management, and functional recovery compared to conventional physical therapy after elbow surgery	“Should not replace active movement or physical therapy”.
Acupuncture (52, 53)	Adjunctive therapy for pain, particularly chronic pain.	“Demonstrated to reduce chronic pain when used alongside traditional analgesia”.	Cost and training are large barriers
Psychological Therapies (CBT, Relaxation) (54, 55)	Holistic adjunct to address pain. Includes relaxation, hypnosis, and Cognitive Behavioral Therapy (CBT).	Leads to improved patient self-efficacy and pain coping skills; meta-analyses show reductions in long-term pain severity and disability	Time consuming, longer length to desired outcome, cost/insurance
Cryoneurolysis (56)	An “emerging modality”. A “novel, ultrasound-guided treatment” that delivers extreme cold to specific nerves.	Provides pain relief.	As a “novel” technique, it is less established than other modalities.

**TABLE 3:** Techniques for objective measurement of pain. This table compares emerging technologies and methodologies for objective pain assessment, noting their validation status and potential clinical utility.

Technique	Mechanism of action	Key considerations
Wearable Forehead Sensor (100)	Measures changes in cerebral hemodynamics via cerebral optical spectrometry	Correlates with patient-report pains scores
Functional Near-Infrared Spectroscopy (99)	Measures changes is oxygenated and deoxygenated hemoglobin in the brain	Requires high level of training to interpret results
Electroencephalogram (EEG) and functional MRI (101)	EEG can detect neural activity associated with pain, while fMRI allows for visualization of key neurologic structures for specific analgesics	EGs and fMRI are both time-intensive procedures that can cost a substantial amount of money
Pupillometry (98)	Measures fluctuations in pupillary diameter	Shown strong correlation in reported pain levels postoperatively

## Abbreviations

ERAS: enhanced recovery after surgery
NSAIDs: non-steroidal anti-inflammatory drugs
ASA: American society of anesthesiologists
MMPM: multimodal pain management
OSA: opioid-sparing analgesia
OFA: opioid-free analgesia
TCAs: tricyclic antidepressants
SNRIs: selective norepinephrine reuptake inhibitors
PONV: postoperative nausea and vomiting
NPRS: numerical pain rating scale
VAS: visual analog scale
EEG: electroencephalogram
fMRI: functional magnetic resonance imaging
